# Passively mode locked thulium and thulium/holmium doped fiber lasers using MXene Nb_2_C coated microfiber

**DOI:** 10.1038/s41598-021-90978-x

**Published:** 2021-06-02

**Authors:** H. Ahmad, R. Ramli, N. N. Ismail, S. N. Aidit, N. Yusoff, M. Z. Samion

**Affiliations:** 1 Photonics Research Centre, University of Malaya, 50603 Kuala Lumpur, Malaysia; 2 Physics Dept, Faculty of Science, University of Malaya, 50603 Kuala Lumpur, Malaysia

**Keywords:** Optical physics, Ultrafast photonics

## Abstract

As a result of the emergence of two-dimensional (2D) materials for various opto-electronics applications, a new class of materials named MXenes have been attracting interests due to their outstanding nonlinear properties. In this work, an MXene niobium carbide (Nb_2_C) was proposed and demonstrated as a saturable absorber to induce mode-locking in thulium- and thulium/holmium-doped fiber lasers. The Nb_2_C solution was first prepared using the liquid exfoliation technique, and then deposited onto a microfiber for integration into the laser cavity. Stable mode-locking operation was observed in both laser cavities, where the center wavelengths of the laser were recorded at 1944 nm for the TDFL and 1950 nm for the THDFL. The generated pulses in the TDFL and THDFL had repetition rates of 9.35 and 11.76 MHz respectively, while their corresponding pulse widths were 1.67 and 1.34 ps. Both of the lasers were highly stable, having SNR values of more than 52 dB and showed no major fluctuations when tested for their long-term stabilities. The results demonstrate an excellent performance of the Nb_2_C as a saturable absorber, offering opportunities to further explore MXenes for future photonics devices.

## Introduction

Historically, in past few decades, the discovery of laser technology has open opportunities that transforms fundamental research to applied research that benefits to the society in wide range of fields to ease and improve their livelihood. The early development of laser device and amplifiers was using a wavelength of 1.55 µm (C-band)^1,2^, applied in global telecommunications networks. Current research has advanced into a new niche area of 1.0 µm^3,4^, 1.46 µm to 1.53 µm (S-band)^5–8^ and 1.565 µm to 1.625 µm (L-band)^9–11^, to meet the demand for higher bandwidth devices. During this time also, generation of various lasers configuration including multiwavelength^12–15^, pulsed^16–21^, swept sources^22–24^ and fiber sensors^25,26^ which cover a broad wavelength range from S- to L-bands has been a priority in various laboratories globally. In addition, 2 µm wavelength lasers can be applied to other fields such as optical spectroscopy, material processing, surgery, light detection and ranging measurements (LIDAR)^27–30^. The main reason for such interest in pursuing studies in 2.0 µm wavelength is due the properties of atmospheric transparency window at around 2.0–2.5 µm wavelength^31–33^. Additionally, a key aspect of 2.0 µm light sources is its ability to be strongly absorbed in the human eye’s vitreous part, reducing the possibility to harm the retina^34^. For an example, a strong water absorption peak at 2.0 µm which makes these lasers useful for specific medical diagnostic and laser surgery devices. 2.0 µm fiber laser sources can be generated from thulium-doped^35^ and thulium/holmium-doped fiber lasers^36^. The thulium and thulium/holmium doped fiber exhibit a broad bandwidth covering almost 500 nm, from 1.7 µm to 2.2 µm. This feature allows a wide selection range of laser operations in the eye-safe spectral region, including the continuous-wave (CW) mode and the Q-switching mode. Such broad bandwidth is also known to be capable of generating femtosecond pulses in the mode-locking regime^37–42^.

Mode-locked fiber lasers as highly versatile light sources have attracted enormous attention due to its ability to access a wide range of scientific and industrial processes including, optical communications, sensing, material processing and medical treatment^43–48^. So far, two main approaches utilized in the operation of mode-locked fiber lasers are based on active and passive techniques. Compared to active, passive technique has the intrinsic advantages of high stability and reproducibility for robust ultrashort optical pulses. Saturable absorber (SA) plays a vital role in passive mode-locking technique. Presently, SA can be classified into two broad types, namely artificial, and real. Artificial SAs, such as nonlinear loop mirrors^49–53^ and nonlinear polarization rotation^54,55^ are based on nonlinear effects with the properties of high damage threshold and low cost. These SAs provide a good platform for operation of high energy laser. However, the vulnerability of the laser system towards environmental perturbation has limited its practical applications. Real SAs, made up of materials that exhibit intensity-dependent transmission are regarded as a more effective way to generate mode-locked pulses. Specifically, two-dimensional (2D) material-based SAs have been widely employed as effective SAs due to their excellent optical properties, including wide absorption band and ultrafast recovery time. Following the successful exfoliation of graphene and its first ultrafast application, other 2D materials including topological insulators (TIs)^41,56–59^, transition metal dichalcogenides (TMDs)^60–65^, metal chalcogenides^66^, antimonene^67^ and MXenes^68^ have been explored for their unique saturable absorption properties in ultrafast laser generation. Additionally, layered transition-metal monochalcogenides has also been used as SA for demonstrating pulsed lasers^69^. In the past few years, Jhon et al*.*^70^ had shown impressive results on 2D materials known as MXenes that could be used as an alternative SA for generation of ultrafast laser.

Transition metal carbides and/or nitrides, which are widely known as MXenes, are a member of the 2D material group that possess unique properties that could be altered by simply manipulating the composition and surface termination elements^71,72^. In general, MXenes consist of few-atoms-thick layers of transition metal carbides, nitrides or carbonitrides with composition of M_n+1_X_n_T_x_, where M stands for an early transition metal (such as: Ti, V, Cr, Nb, etc.), X stands for carbon and/or nitrogen, n = 1, 2, or 3, and T_x_ is the surface termination groups ((–O), (–F), and (–OH))^73^. Being in the family of MXene, niobium carbide (Nb_2_C) has received extensive research attention in the last few years, due to its unique physical and chemical properties that are valuable in various applications^74^. Theoretically, it has been predicted that Nb_2_C demonstrates a great reduction of lattice thermal conductivity resulted from the abnormal electron–phonon scatterings with intensities close to that of phonon–phonon scatterings^75^. In a study conducted by *Lin et. al.*^76^ has revealed that the Nb_2_C possess strong optical response in the near infrared region as it shows high photothermal conversion efficiency that can be used in biomedicine, particularly for cancer phototherapy. In another report, *Wang et. al*.^77^ has investigated the broad-band nonlinear optical response and the ultrafast carrier dynamics of Nb_2_C over the wavelength ranging from visible to the near-infrared region. Their finding disclosed the dependency of the nonlinear optical response of Nb_2_C on wavelength and excitation intensity. The unique nonlinear absorption response inversion properties of Nb_2_C, that is the ability to shift from saturable absorption to two-photon absorption in near infrared region, has facilitated its vast applications in nonlinear photonics, in particular as an optical switch^78^.

In this work, high-quality few layer Nb_2_C nanosheets were fabricated by the liquid phase exfoliation method and deposited onto a microfiber using a drop-casting method, forming an all-fiber SA device. Based on Nb_2_C-coated microfiber SA, passive mode-locking operation in TDFL and THDFL were successfully generated. Both laser systems show stable mode-locked pulses with the former system operating at 1948 nm and the later operating at 1952 nm. These results suggest that the Nb_2_C-coated microfiber could perform as a practical and a high-performance SA for an ultrafast fiber laser generation in the 2.0 µm region. This could further promote the development of MXene-based optical devices in the photonics technology.

### Characterization of Nb_2_C MXene

**Figure 1:**
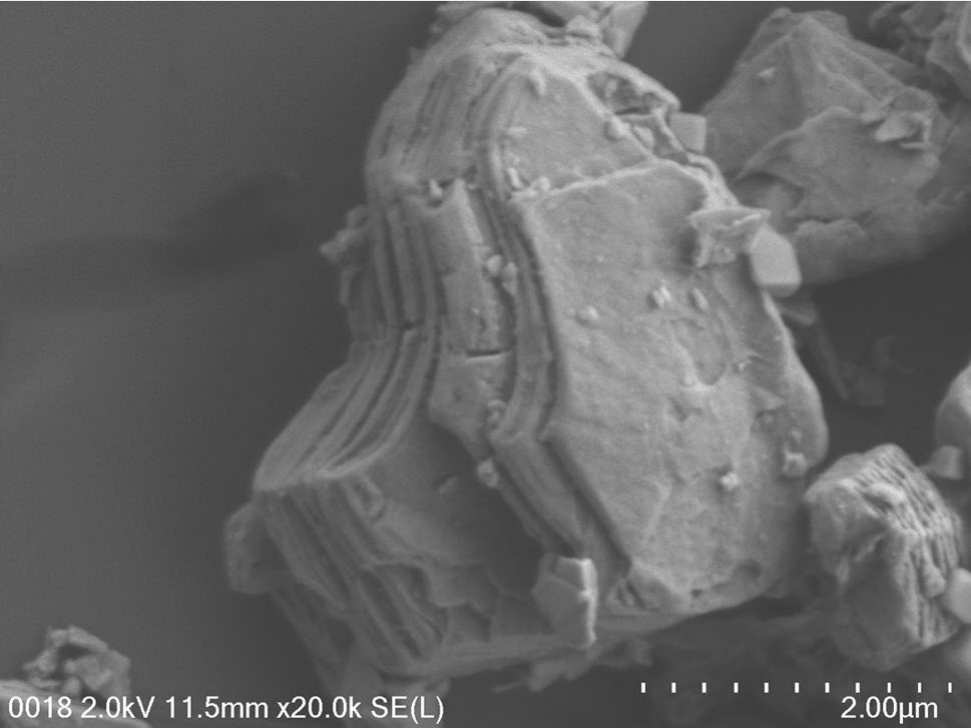
FESEM image of Nb_2_C MXene in the powder form.

The surface morphology of the Nb_2_C MXene in the powder form was investigated by field emission scanning electron microscopy (FESEM) and the elemental composition of Nb_2_C MXene was determined by energy dispersive X-ray (EDX) analysis. These studies were performed using a Hitachi Model SU8220 FESEM which was equipped with EDX detector and operating at 2.0 kV. Figure 1 shows the FESEM image of Nb_2_C Mxene captured at the magnification of 20.0 k. It can be observed that Nb_2_C MXene exhibits a sheet-like structure with multiple layers stacked together, whose size is about 2.0 μm.

**Figure 2:**
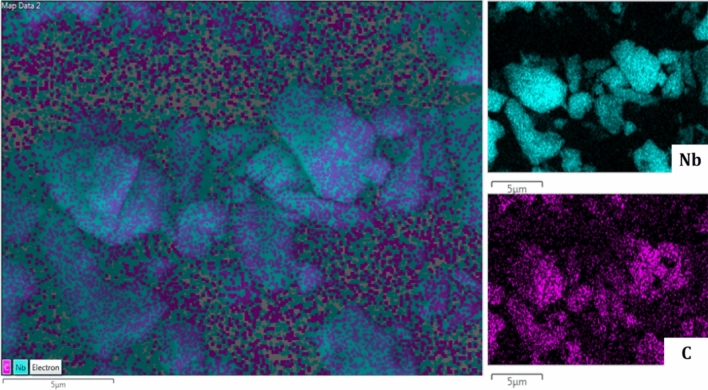
EDX elemental mapping of Nb_2_C MXene.

Figure 2 represents the EDX elemental mapping images of Nb_2_C MXene, where the distribution of each element can be clearly seen. The Niobium (Nb) and Carbon (C) maps verified the presence of Nb and C elements, thus confirming the successfully formation of Nb_2_C MXene.

**Figure 3:**
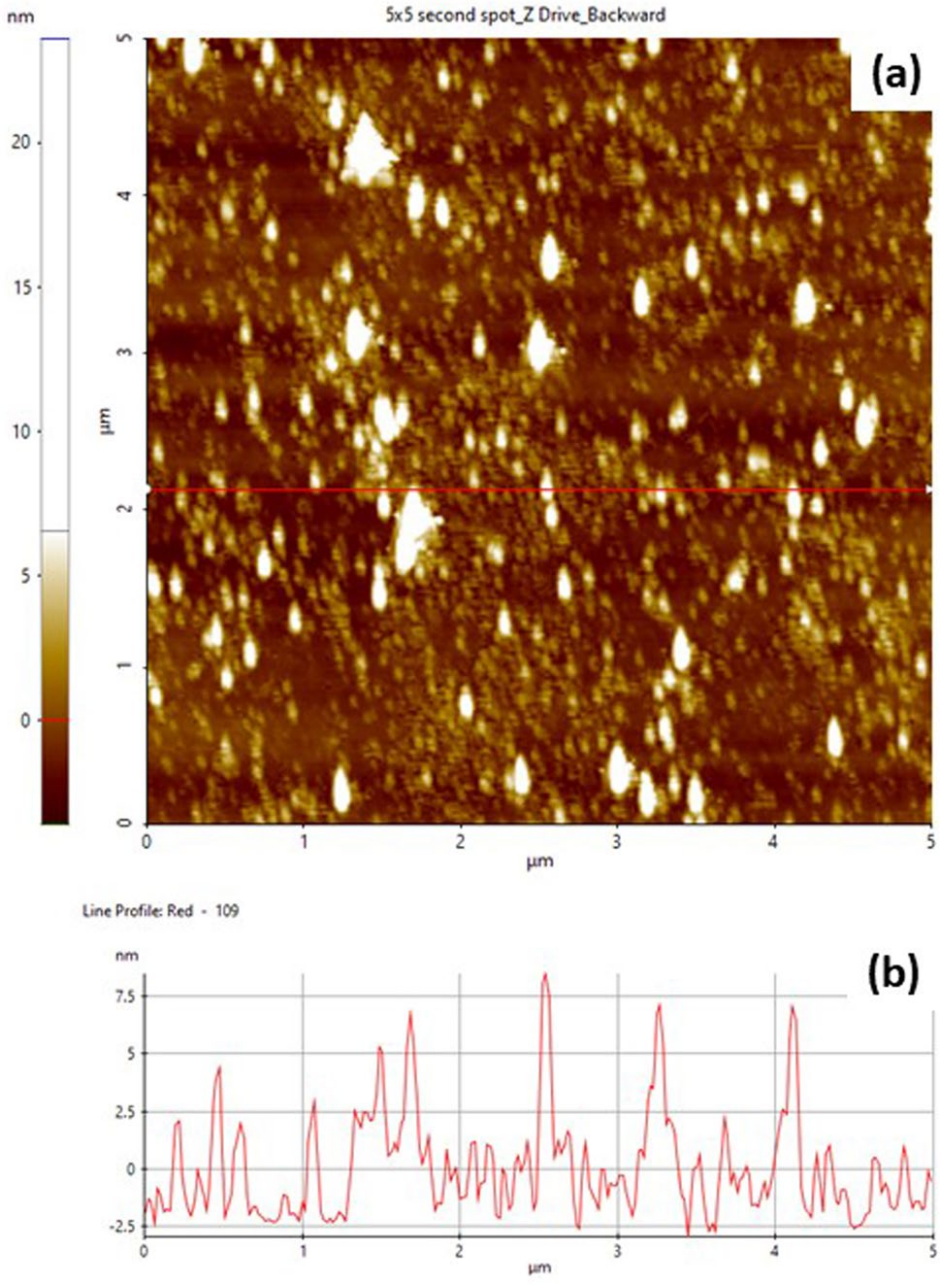
**(a)** AFM topography image of Nb_2_C MXene and **(b)** the corresponding lateral height measurement.

An atomic force microscope (Park System NX-10 AFM) was used to measure the thickness of the Nb_2_C MXene. The measurement was done under non-contact mode. Initially, 10 μL of Nb_2_C MXene solution was deposited on the Si substrate using the spin coating technique. The sample was dried at room temperature overnight before being used for thickness measurement. The AFM topography image of Nb_2_C MXene together with its corresponding lateral height measurement are presented in Fig. 3(a) and Fig. 3(b), respectively. The obtained result demonstrates that the Nb_2_C MXene flakes exhibit different lateral sizes ranging from 20 to 300 nm with the average thickness of about 2.5 nm. The thickness of MXene nanosheets obtained in this work is the typical thickness of MXene reported elsewhere^79,80^.

### Preparation and Characterization of MXene Nb_2_C-coated microfiber SA

**Figure 4:**
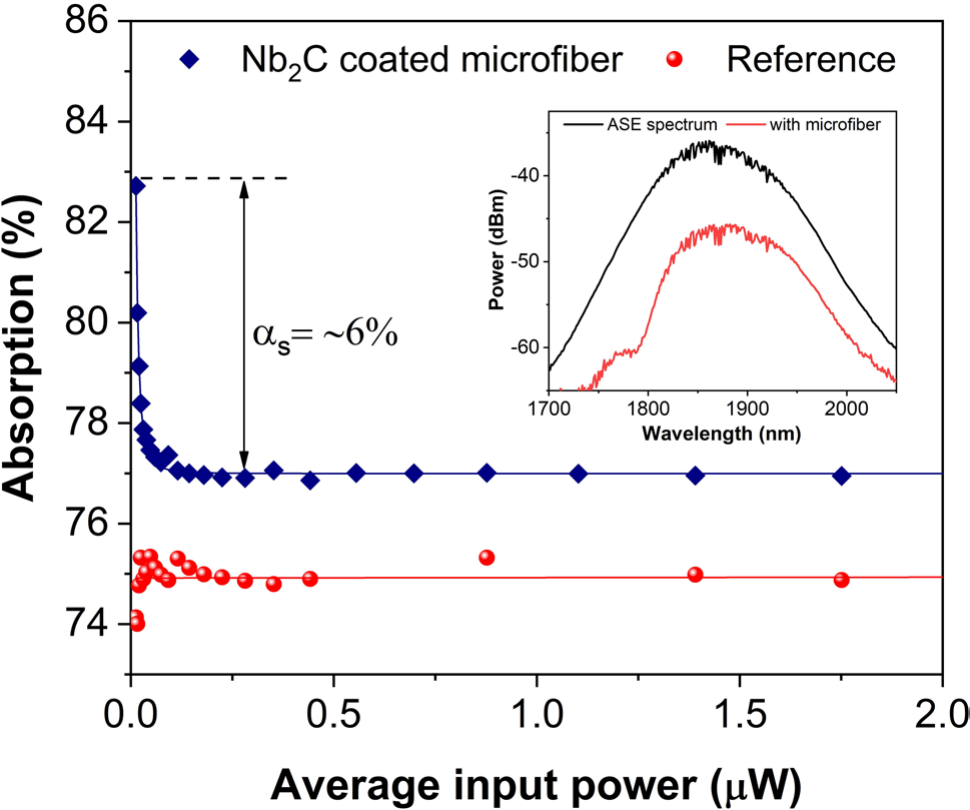
Nonlinear absorption curves of reference sample (without Nb_2_C coating) and Nb_2_C coated microfiber. Inset shows the spectral transmittance of microfiber prior to the Nb_2_C coating.

The measurement of nonlinear optical absorption of MXene Nb_2_C-coated microfiber SA was examined using the twin-detector measurement technique. A 1950 nm Toptica FemtoFerb femtosecond laser with a repetition rate of 30 MHz and a pulse width of 100 fs was employed as the seed laser. The laser source was connected to a variable attenuator and subsequently to a 3 dB optical coupler for beam splitting. One port of the 3 dB coupler was connected to the Nb_2_C-coated microfiber SA and another port was connected directly to a microfiber without the SA as the reference port. Both transmitted powers were measured using optical power meter. Additionally, the saturable absorption of the reference sample which is microfiber without Nb_2_C coating was also studied. The experimental data were recorded and fitted using the saturation model equation below:

$$\alpha (I)= \frac{{\alpha }_{s}} {1+ \raisebox{1ex}{\it I}\!\left/ \!\raisebox{-1ex}{\it I} _{sat} \right.} {+ {\alpha }_{ns}}$$

where *I*, *I*_*sat*_, α_*ns*_, α_s_ signify the laser input intensity, saturation intensity, non-saturated loss and modulation depth. Nonlinear absorption curves of the reference sample (without Nb_2_C coating) and Nb_2_C coated microfiber are presented in Fig. 4. The power dependent loss of the reference sample was first studied. It can be seen that the absorption loss remains constant with increasing pump powers, thus eliminating saturable absorption or any other optical phenomena in the microfiber itself. The insertion loss of this device was approximately 75% (6 dB), slightly lower compared to ~ 82.5% (~7.5 dB) insertion loss of microfiber coated with Nb_2_C. On the contrary, the saturation intensity and modulation depth of the Nb_2_C coated microfiber were obtained around 0.016 µW and 6%, respectively. The value of 0.016 µW corresponds to a power intensity of 0.4 MW/cm^2^, which was calculated by dividing the average power with its repetition rate, then the pulse width and also by taking the core diameter of the SMF to be 9 µm.

### Experimental setup

**Figure 5:**
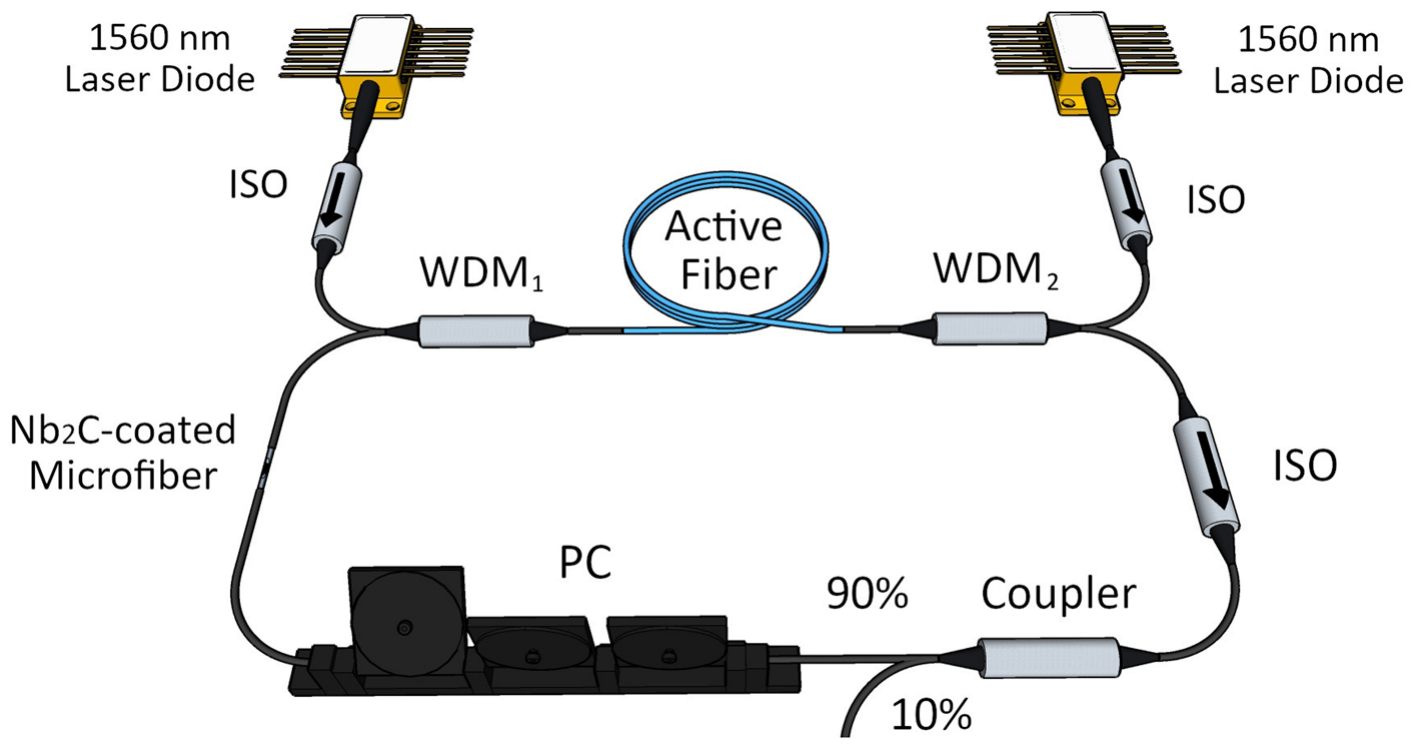
Schematic diagram of 2.0 µm passively mode-locked fiber laser cavity. This figure was drawn using SketchUp Make 2017 (Basic), Software Version: Windows 64-bit 17.2.2555, available at https://www.sketchup.com/download/all.

To prepare the SA device, the Nb_2_C solution was drop-casted onto a microfiber (tapered fiber) with a waist diameter and tapered length of around 8 µm and 3 cm, respectively. The insertion loss of the microfiber at 2000 nm was initially measured to be 6 dB without the Nb_2_C, while the value increased to 7.5 dB after the material was deposited. A high microfiber insertion loss prior to the Nb_2_C coating could be attributed to a strong evanescent field and possible water absorption at 2000 nm. At the 2000 nm wavelength region, the mode field diameter (MFD) of the propagating signal in the optical fiber is larger compared to the 1550 nm wavelength^81^. A larger MFD value means a higher percentage of the optical power propagating in the cladding region. When the diameter of the fiber is reduced, the propagating optical power in the cladding will be guided in the air-cladding interface and it could not be recaptured by the core, resulting in the loss of power. The transmittance spectral of the microfiber without Nb_2_C is shown in the inset of Fig. 4. The transmission spectrum is the same as the input spectrum but reduced in power, indicating the adiabatic nature of the microfiber and it shows no interaction with higher order modes. It must be noted that the visible dips observed in both spectra are due to the strong water absorption lines at 1.9 µm region. The Nb_2_C-coated microfiber was incorporated into the THDFL and TDFL cavities that utilized the dual pumping scheme as shown in Fig. 5. Both lasers had the same cavity design, except for the different active fibers being used as the amplifying medium. Two laser diodes (LDs) with center wavelengths at 1560 nm were used as the pump source to pump the active fiber. Each LD was then connected to a 1550 nm polarization insensitive isolator (PI-IO) to protect the LD from back-reflections. A 1550/2000 nm wavelength division multiplexer (WDM) was placed after each of the 1550 nm PI-ISO to guide the 1560 nm pump light to the active fiber. A 2000 nm PI-ISO was placed after WDM_2_ to allow the light to only propagate in the clockwise direction. The output of the PI-ISO was then connected to a 90:10 coupler, where 10% of the signal was taken out as the output whereas the 90% was looped back to the cavity. A polarization controller (PC) was connected to the 90% port of the coupler and was used to adjust the polarization states of the circulating light. The other end of the PC was connected to the Nb_2_C-coated microfiber, then to the 2000 nm port of the first WDM_1._ Thus, completing the ring laser cavity.

The total length of the TDFL was measured around 22.1 m, consisting of 4 m of the TDF and 18.1 m single mode fiber (SMF-28), whereas the THDFL had a total cavity length of 17.4 m, involving of 1.5 m of the THDF and 15.9 m of SMF-28. The material dispersion values of TDF (TmDF200, OFS) and THDF (TH512, CorActive) were 10.89 ps nm^−1^ km^−1^ at 1944 nm and 33.4 ps nm^−1^ km^−1^ at 1950 nm, respectively. These values were given by the manufacturer. The material dispersion for SMF-28 was calculated using the formula given by Corning and was based on the SMF-28 datasheet. The material dispersion for SMF-28 was calculated to be 32.95 and 33.31 ps nm^−1^ km^−1^ at the laser wavelengths of 1944 and 1950 nm, respectively. The group velocity dispersions (GVDs) of the TDF and THDF were then calculated using the equation $GVD={-\lambda }^{2}\cdot {D}_{\lambda }/2\pi\mathrm{c}$, whereby λ is the center wavelength, D_λ_ is the material dispersion and c is the speed of light. Hence, the calculated GVDs of the TDF and THDF were −0.0222 ps^2^ m^−1^ and −0.0675 ps^2^ m^−1^, respectively. The GVD of SMF-28 was -0.0663 ps^2^ m^−1^ at 1944 nm and −0.0672 ps^2^ m^−1^ at 1950 nm. The calculated GVDs for the active fibers (TDF and THDF) and SMF−28 are in agreement with previous study^82–84^. By computing the ${L}_{SMF-28}{GVD}_{SMF-28}+{L}_{Active fiber}{GVD}_{Active fiber}$, the net cavity dispersion of −1.28 ps^2^ and −1.17 ps^2^ were obtained for TDFL and THDFL, respectively, indicating that the operation of both lasers were in the anomalous dispersion regime. The characteristics for the gain medium for both cavities are given in Table 1.

**Table 1:** Characteristics of the active fibers used in the TDFL and THDFL cavities.

Cavity	Thulium-doped Fiber Laser	Thulium/Holmium-doped Fiber Laser
Active fiber	OFS Thulium doped fiber (TmDF200)	CorActive Thulium/Holmium doped fiber (TH512)
Length	4 m	1.5 m
Numerical aperture	0.26	0.16
Absorption	22 dB/m at 1550 nm	15 dB/m at 1550 nm
GVD	−0.0222 ps^2^/m	−0.0675 ps^2^/m

## Results and discussion

### Mode-locked Thulium-doped fiber laser (TDFL) using Nb_2_C

**Figure 6:**
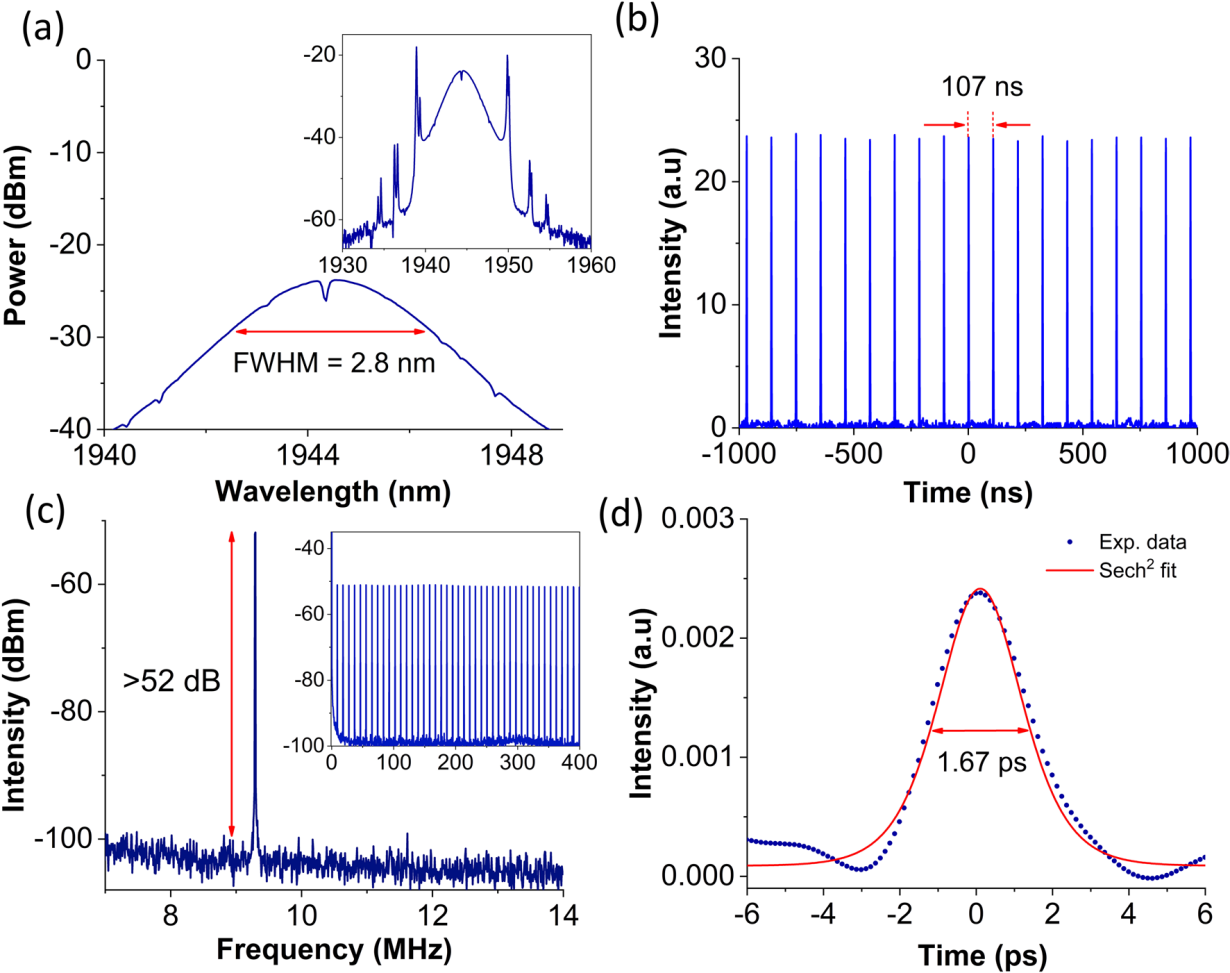
Mode-locked TDFL output at 476 mW. **(a)** Enlarged optical spectrum (Inset shows full soliton spectrum); **(b)** oscilloscope trace; **(c)** radio frequency (RF) spectrum (Inset shows the RF spectrum at a wider span range of 400 MHz); and **(d)** autocorrelation trace.

The performance of the Nb_2_C coated microfiber for the generation of mode-locking was first studied using the TDFL cavity. Mode-locked laser was initiated at pump power of 123 mW, with a suitable adjustment of PC. Figure 6 summarizes the characteristics of mode-locked TDFL at pump power of 476 mW. The soliton spectrum, which is a typical spectrum of mode-locked fiber laser operating in the anomalous regime, is depicted in Fig. 6(a). The minor dip observed near the center of the spectrum at around 1944 nm could be originating from the strong water absorption in 1.9 µm region^85^. This eliminates the possibility of soliton molecules in the fiber laser^56,86^. The double Kelly sidebands observed in the spectrum could be an indication that the mode-locked laser was a group-velocity-locked-vector (GVLV) soliton, and that the double Kelly sidebands originated from the different polarization states of GVLV soliton^87^. The spectrum was centered at 1944 nm with a 3-dB bandwidth of 2.8 nm. The mode-locked pulse train is depicted in Fig. 6(b), having a repetition rate of 9.35 MHz. The pulse train exhibits a uniform pulse intensity with a pulse interval of 107 ns, which augurs well with the cavity round-trip time. Figure 6(c) shows the RF spectrum with a resolution bandwidth of 1 kHz. A distinct and sharp peak was observed at 9.35 MHz with signal-to-noise ratio of more than 52 dB. Figure 6(d) shows the autocorrelation trace fitted by a hyperbolic-secant (Sech^2^) function, showing a pulse duration of 1.67 ps. With values obtained from the mode-locked, the computed time bandwidth product (TBP) is 0.320 which is marginally higher than 0.315 indicating that the pulse is slightly chirped.

### Mode-locked Thulium/holmium-doped fiber laser (THDFL) using Nb_2_C

**Figure 7:**
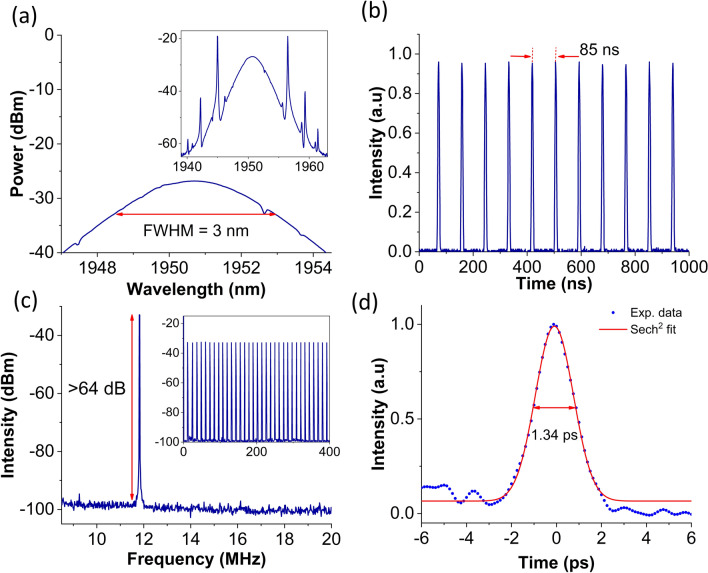
Mode-locked THDFL output at 397 mW. **(a)** Enlarged optical spectrum (Inset shows full soliton spectrum); **(b)** pulse train; **(c)** radio frequency (RF) spectrum (Inset shows RF spectrum over a wider span range of 400 MHz); and **(d)** autocorrelation trace.

Subsequently, the Nb_2_C coated microfiber was incorporated into the THDFL. The properties of the mode-locked THDFL is demonstrated in Fig. 7 at pump power of 397 mW. Figure 7(a) illustrates the mode-locked THDFL spectrum, having a center wavelength at 1950.8 nm with a full-width at half maximum (FWHM) of 3 nm. Similar to the output spectrum of the TDFL, Kelly sidebands were also observed in the optical spectrum. This was the result of the gain fibers exhibiting a relatively large anomalous dispersion in the 2.0 µm region, causing the mode-locking operation of the 2.0 µm fiber lasers often lying in the conventional soliton regime. Figure 7(b) shows the mode-locked pulse train having a pulse interval of 85 ns, with a corresponding repetition rate of 11.76 MHz. The RF spectrum was also measured and shown in Fig. 7(c). A distinct peak was observed at the frequency of 11.76 MHz with an SNR of more than 64 dB. Additionally, the RF spectrum over a wider range of 400 MHz shown in the inset of Fig. 7(c) was seen to be free from any spectral modulations, indicating the absence of possible Q-switching instabilities. The pulse profile obtained from the autocorrelation measurement was fitted with a sech^2^ profile, as illustrated in Fig. 7(d). The observed side bumps observed in our work resulted from the noise floor of the detection system instead of the optical signal^88^. Mode-locked laser operating in the soliton bound regime typically exhibits side-bumps in the autocorrelation (AC) trace with intensities as high as half of the intensity of the main peak^87^. In the AC trace of both of the TDFL and THDFL, the intensities of the side-bumps were insignificant. Hence, the unsymmetrical side humps with insignificant intensities exclude the possibility of a bound soliton operation. The pulse width verified with fitted Sech^2^ function is around 1.34 ps. The TBP was estimated to be 0.317, which was only slightly larger than the TBP limit of 0.315. This indicates that the generated pulses were close to their transform-limited operation.

**Figure 8:**
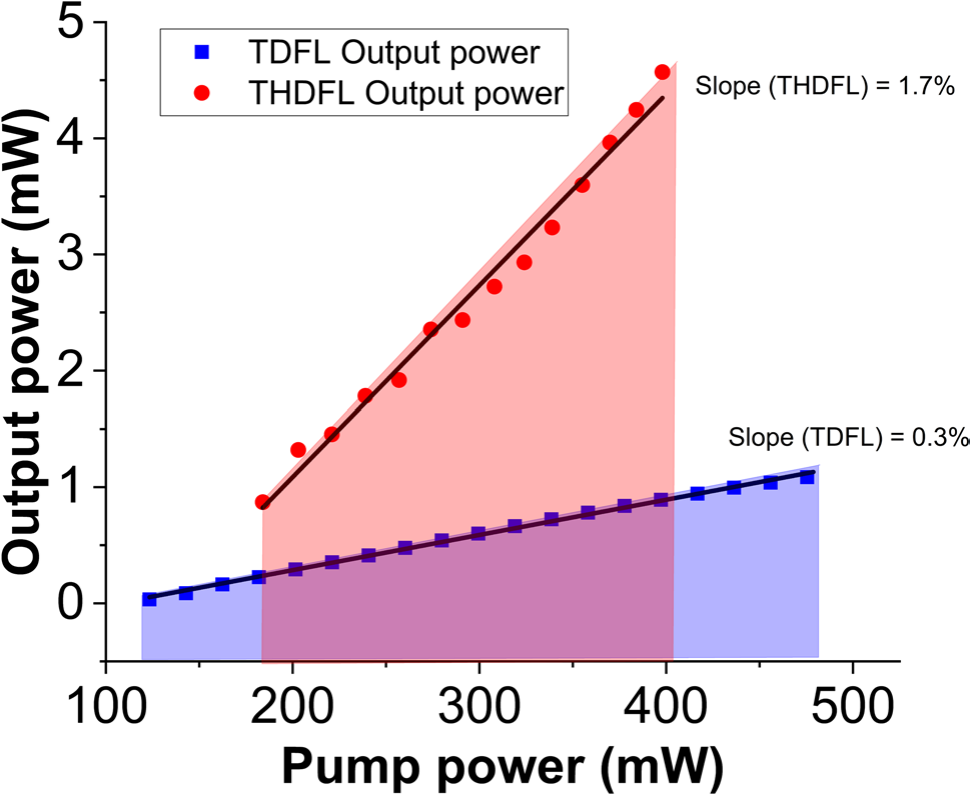
Variation of output power against pump power for TDFL and THDFL.

Figure 8 shows the variation of average output power against pump power for both mode-locked TDFL and THDFL cavities. Low mode-locking threshold of 123 mW was observed in the TDFL. At the maximum pump power of 476 mW, the maximum output power for the mode-locked TDFL was recorded to be around 1.1 mW. The slope efficiency of the TDFL was 0.3%. The corresponding pulse energy and peak power were calculated to be around 0.12 nJ and 70 W, respectively. Meanwhile, the mode-locked THDFL had a maximum output power of around 4.6 mW at maximum pump power of 397 mW with a corresponding pulse energy of 0.39 nJ and a peak power of 291 W. As compared to the TDFL, a higher slope efficiency of 1.7% was obtained in the THDFL. Both output powers could be linearly fitted with minimal error, and indicate that both lasers are capable of generating higher output powers if higher pump powers are available. According to the soliton energy theorem, the maximum pulse energy generated in the mode-locked laser cavity is quantized^89^. At higher pump power levels, the pulse circulating in the laser cavity could split into several pulses. However, no pulse splitting was observed in our work within the recorded pump power range, which may be due to the limitation of our current measurements that could not resolved the pulses. We believe that further investigation would be needed and will be of interest in future studies.

**Figure 9:**
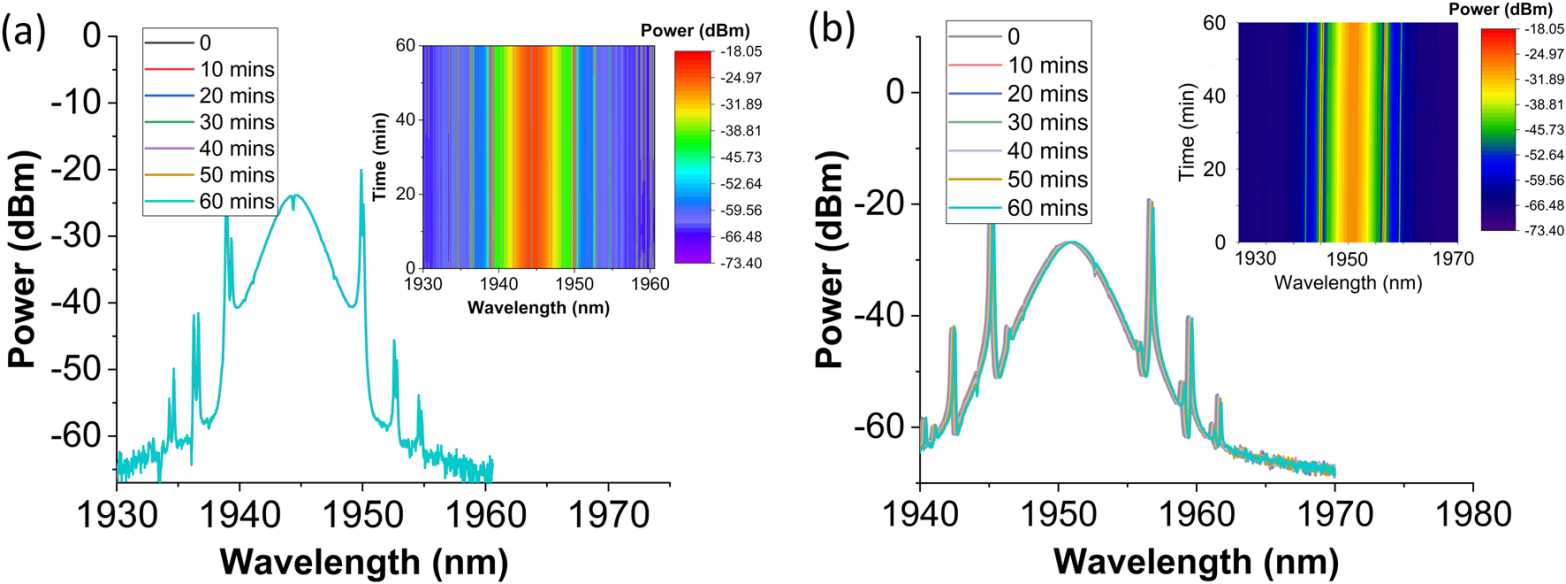
Stability measurement of **(a)** TDFL at 476 mW and **(b)** THDFL at 397 mW over a period of 1-h.

The Nb_2_C-coated microfiber SA also exhibited an excellent stability against degradation, maintaining stable mode-locking operation when tested for its long-term stability. This can be seen from Fig. 9, where the mode-locked spectrum of TDFL and THDFL was recorded at every 10 min for 1-h. Throughout the stability measurement, the center wavelengths of the lasers were maintained at 1944 nm and 1950.8 nm for the TDFL and the THDFL, respectively. Additionally, no significant power fluctuation was observed during their operations. Interestingly, the mode-locked pulse laser can still be achieved even though the SA has been left for few weeks since the first result was obtained. This shows that the fabricated Nb_2_C-coated microfiber SA exhibits long term stability. To further verify the generation of mode-locked pulses in both laser cavities were induced by Nb_2_C-coated microfiber SA, the SA device was removed from the laser cavities. At any pump power level, only continuous-wave (CW) laser operation was observed in both TDFL and THDFL. This proves that the mode-locking operation was induced by the saturable absorption effect from the Nb_2_C completely.

Table 2 shows the comparison of the output performance of mode-locked pulse laser with the used of different types of MXene as SA. The earliest pulse generation research was done by Jhon et al*.*^70^ which uses Ti_3_CN drop-casted onto a side-polished fiber to produce mode-locked pulses with a central wavelength, pulse width and repetition rate of 1557 nm, 660 fs and 15.4 MHz respectively in an EDF cavity. Jiang et al*.*^90^ explored a new MXene Ti_3_C_2_T_x_ with two wavelengths region of 1065 nm and 1555 nm using a YDF and EDF respectively. With the same material, Li et al*.*^91^ was able to produce stable mode locked pulses in an EDF cavity with a longer wavelength of 1567 nm using a side polished fiber. Of late, Huang et al*.*^92^ and Ma et al*.*^93^ had used V_2_CT_x_ coated on the microfiber as an SA to produce pulsed laser with pulse width of 3.21 ps and 2.3 ps in EDF and YDF cavity, respectively. The above-mentioned findings using gain medium of YDF and EDF showed that the pulse laser with MXene as a SA could be generated in the region of 1.0 μm to 1.5 μm^70,90–93^. Previously, Gao et al*.*^74^ reported that pulsed laser was generated by MXene Nb_2_C in Thulium-doped fiber (TDF) laser using a microfiber with the central wavelength values of 1889 nm and pulse width of 2.27 ps. Correspondingly, the results of our research showed better mode-locked characteristics in terms of a longer wavelength (1944 nm) and narrower pulse width (1.67 ps) which may have better potential applications in pulsed laser technologies. Ahmad et al*.* (using TDF and HDF laser cavity)^94,95^ showed the generation of Q-switched lasers with wavelength outputs of 1976 nm and 2079 nm, respectively. However, our current findings could generate mode-locked lasers with wavelength output of 1944 nm (TDF) and 1950 nm (THDF), which we believe to be the first to be reported in the literature. On top of this, the related pulse width characteristics were 1.67 ps (TDF) and 1.34 ps (THDF), indicating a narrower output than those obtained in previous works [4.4 μs to 2.27 ps]^74,92–95^. Consequently, using MXene Nb_2_C with TDFL and THDFL may provide the inroads to generate ultrafast lasers in 1.9 to 2.0 μm region.

**Table 2:** Comparison of pulsed laser characteristics with MXene as SA.

Material (MXene)	Laser	Gain medium	Central wavelength	Pulse width	Repetition rate	Configuration	Ref
Ti_3_CN	Mode-locked	EDF	1557 nm	660 fs	15.4 MHz	Side-polished fiber	^70^
Ti_3_C_2_T_x_	Mode-locked	YDF	1065 nm	480 fs	18.96 MHz	Microfiber	^90^
Ti_3_C_2_T_x_	Mode-locked	EDF	1555 nm	159 fs	7.28 MHz	Microfiber	^90^
Ti_3_C_2_T_x_	Mode-locked	EDF	1567 nm	946 fs	8.24 MHz	Side polished fiber	^91^
V_2_CT_x_	Mode-locked	EDF	1559 nm	3.21 ps	4.9 MHz	Microfiber	^92^
V_2_CT_x_	Mode-locked	YDF	N.A	2.3 ps	N.A	Microfiber	^93^
Nb_2_CT_*x*_	Mode-locked	TDF	1889 nm	2.27 ps	6.28 MHz	Microfiber	^74^
Nb_2_C	Q-switched	HDF	2079 nm	4.4 μs	20.5 kHz	Thin film	^94^
Ti_3_C_2_T_x_	Q-switched	TDF	1976 nm	2.4 μs	59 kHz	Microfiber	^95^
**Nb**_**2**_**C**	**Mode-locked**	**TDF**	**1944 nm**	**1.67 ps**	**9.35 MHz**	**Microfiber**	**This work**
**Nb**_**2**_**C**	**Mode-locked**	**THDF**	**1950 nm**	**1.34 ps**	**11.76 MHz**	**Microfiber**	**This work**

*N.A.: Not available in the paper.

The bold in the table is to highlight the results from this work.

## Methods

### Preparation of Nb_2_C MXene

Initially, 50 mg of Nb_2_C powder (2D Semiconductors, USA) was added in a glass vial containing 10 mL of isopropyl alcohol (IPA, Sigma Aldrich). The mixture was put under the sonication process using a Hielscher UP200Ht probe type sonicator to obtain a homogenous solution. The sonicator was operated with a 5 s on pulse, 5 s off pulse and 40% power for 4 h. Next, the obtained black colour suspension was centrifuged for 10 min at 4000 rpm to separate the undissolved powder. The supernatant was collected for further use and has been labelled as Nb_2_C Mxene solution.

### Laser characterisation

To analyse the output mode-locked pulses, a 2 µm Newport 818-BB-51F photodetector was used together with a Yogokawa DLM2054 oscilloscope and a Rohde & Schwarz FPC1000 radio-frequency spectrum analyser (RFSA). The spectrum of the mode-locked laser was recorded using a Yokogawa AQ6375 optical spectrum analyser (OSA) at the highest resolution of 0.05 nm. The pulse width measurement was measured by an A.P.E Pulsecheck 150 autocorrelator while the output power was measured using a Thorlabs S148C optical power meter (OPM).

## Conclusion

An MXene niobium carbide (Nb_2_C) was successfully proven as a saturable absorber to produce stable mode-locked pulses in the 2.0 µm wavelength region. The Nb_2_C solution was coated onto a microfiber by the drop-cast method to allow easy integration into thulium- and thulium/holmium-doped fiber laser cavities. The TDFL had a center wavelength at 1944 nm, while the center wavelength of the THDFL was recorded at a slightly longer wavelength at 1950 nm. The generated pulses in the TDFL and THDFL had repetition rates of 9.35 and 11.76 MHz, respectively, while their corresponding pulse widths were 1.67 and 1.34 ps. The maximum peak power generated was 70 W for the TDFL and as high as 291 W for the THDFL. The lasers were highly stable when tested for their long-term stabilities, where no major fluctuation in the center wavelength as well as the peak optical power was observed. The stability was further proven with the SNR values of both lasers recorded to be more than 52 dB. The results show the use of the Nb_2_C as a promising mode-locker, offering opportunities to further explore the use of MXenes for future photonics devices.
